# Improving accuracy of SUV estimates in paediatric oncology: Recommending against the use of body weight corrected SUV in [^18^F]FDG PET

**DOI:** 10.1007/s00259-025-07104-6

**Published:** 2025-02-08

**Authors:** Isabelle S. A. de Vries, Silke Lodema, Arthur J. A. T. Braat, Johannes H. M. Merks, Rob van Rooij, Bart de Keizer

**Affiliations:** 1 Princess Máxima Center for Pediatric Oncology, Utrecht, The Netherlands; 2 Division of Imaging and Oncology, University Medical Center Utrecht, Utrecht University, Utrecht, The Netherlands

**Keywords:** [^18^F]FDG PET/CT, Standardized uptake value, Paediatric patients, Body weight

## Abstract

**Purpose:**

Few studies have assessed body weight dependency of Standardised Uptake Value (SUV) formulations in paediatric patients. This study aims to compare different SUV formulations measured in reference tissues in paediatric patients and determine which correction method shows the least dependency on body weight.

**Methods:**

A single-centre, retrospective analysis of [18F]FDG PET/CT scans was performed. SUV measurements were taken from liver and blood pool using EARL1 reconstructions. SUV measurements were corrected for body weight (SUVBW), lean body mass (LBM) according to James (SUVLBMJames) and Janmahasatian (SUVLBMJanma), and body surface area (BSA) according to DuBois (SUVBSADuBois) and Haycock (SUVBSAHaycock). The coefficient of determination (r^2^) was used to assess the correlation between SUV and body weight.

**Results:**

In total, 461 scans were analysed, including 185 (40%) from female patients. The median age of patients was 12 years (IQR 8–15.5 years). SUVBW exhibited the strongest correlation with body weight, with r^2^ = 0.65 for the liver and r^2^ = 0.50 for the blood pool. In contrast, SUVBSADuBois and SUVBSAHaycock had the weakest correlation, with r^2^ = 0.09 for the liver and r^2^ = 0.06 for the blood pool. SUVLBMJames and SUVLBMJanma had moderate correlations, with r^2^ = 0.51 and r^2^ = 0.44 for the liver and blood pool, respectively, and r^2^ = 0.47 and r^2^ = 0.42, respectively.

**Conclusion:**

In paediatric [18F]FDG PET/CT scans, SUVBW should be avoided due to elevating values in heavier patients. SUVBSA presents the least dependency on body weight and provides the most consistent assessments of metabolic activity.

**Supplementary Information:**

The online version contains supplementary material available at 10.1007/s00259-025-07104-6.

## Introduction

Standardised Uptake Value (SUV) measurements are widely used to quantify [^18^F]FDG uptake in PET/CT imaging, with the SUV calculated as the ratio of radioactivity concentration in a specific region of interest (ROI) to the average activity concentration in the patient’s body. Traditionally, the average activity concentration is estimated by dividing the injected radiotracer activity by the patient’s body weight, which is assumed to reflect the volume of distribution. However, since adipose tissue has minimal [^18^F]FDG uptake, body weight may not provide an accurate estimate of distribution volume, particularly in obese patients [1, 2].

To address this limitation, several alternative SUV formulations have been investigated in adults to account for variations in body composition. These include corrections based on lean body mass (LBM) and body surface area (BSA). Notable formulations for LBM include the James (LBMJames) [3] and Janmahasatian (LBMJanma) [4] formulas, while BSA corrections are commonly made using the DuBois (BSADuBois) [5] and Haycock (BSAHaycock) [6] formulas. Studies have shown that for adults, these adjustments produce in SUV measurements that are less dependent on body weight than the traditionally body weight corrected SUV [1, 2, 7–10].

While the relationship between body weight and SUV has been well-studied in adults, it is crucial to understand this relationship in children, whose body composition undergoes significant changes as they grow. Additionally, in paediatric oncology patients, treatments can lead to significant fluctuations in body weight [11–13]. Such variations complicate the comparability of SUV measurements both within individual patients over time and across different patient groups. Given these challenges, there is a need to evaluate which SUV formulations offer the most reliable and body weight-independent measurements in paediatric populations.

This study aims to compare different SUV formulations in paediatric oncology patients by analysing reference tissues such as the liver and blood pool, which exhibit stable glucose uptake [14]. Through this analysis, we seek to determine which SUV formulation minimises the dependency on body weight, offering more accurate and consistent measurements for paediatric PET/CT imaging.

## Material and methods

### Study design and setting

This is a retrospective, single centre, cross-sectional study including paediatric oncology patients aged 0 to 18 years at diagnosis, who underwent whole-body [^18^F]FDG PET/CT scans between December 2018 and August 2022. If multiple scans were available, the first scan of each patient within this timeframe was included. Patients with liver tumours were excluded due to the potential influence of tumour metabolism on the reference SUV measurement in the liver.

### Image acquisition

All scans were performed using a Biograph Vision 600 PET/CT system (Siemens Healthineers) [15]. [^18^F]FDG PET/CT scans were performed per EARL protocol [16]. Patients fasted for at least 6 h before scanning, and whole-body imaging was performed 60 min after the intravenous injection of 2.0 Megabecquerel (MBq)/kg [^18^F]FDG, with a minimum of 20 MBq administered. A low-dose CT scan without contrast enhancement was used for attenuation correction (120 kV, 40 mAs). PET images were reconstructed using an OSEM 3-dimensional reconstruction applying time-of-flight, with 4 iterations and 5 ordered subsets after attenuation correction. A gaussian filter of 6.0 mm was applied. A matrix size of 220 × 220 was used with a pixel size of 3.3 mm × 3.3 mm and a slice thickness of 3 mm. The scan speed was 1.6 mm/second. Patient height was measured in the outpatient clinic prior to the scan, and body weight was recorded in the scanning room.

### Study parameters

Two researchers (IV and SL) performed the measurements on the [^18^F]FDG PET/CT scans using the EARL1 reconstructions and thin-slice CT series. RadiAnt DICOM viewer 2023.1 software (Medixant, Poland) was used to conduct the measurements. SUVmean values for liver and blood pool were measured by manually placing a ROI of 10 cm^2^ in the liver and 0.5 cm^2^ in the aorta. SUVmean was defined as mean uptake of all voxels within the selected ROI. Measured SUVBW was used to calculate SUVLBMJames, SUVLBMJanma, SUVBSADuBois, and SUVBSAHaycock [6] (Table 1).

**Table 1:** Equations of the different SUV body composition corrections

SUV body composition correction	
SUVBW	$$\frac{Radioactive concentration in tissue}{\frac{injected dose}{body weight}}$$
SUVLBMJames Female	$$\frac{Radioactive concentration in tissue}{\frac{injected dose}{1.07*body weight-148*{\left(\frac{body weight}{height}\right)}^{2}}}$$
SUVLBMJames Male	$$\frac{Radioactive concentration in tissue}{\frac{injected dose}{1.1*body weight-128*{\left(\frac{body weight}{height}\right)}^{2}}}$$
SUVLBMJanma Female	$$\frac{Radioactive concentration in tissue}{\frac{injected dose}{\frac{9.27*{10}^{3}*body weight}{8.78*{10}^{3}+244*BMI}}}$$
SUVLBMJanma Male	$$\frac{Radioactive concentration in tissue}{\frac{injected dose}{\frac{9.27*{10}^{3}*body weight}{6.68*{10}^{3}+216*BMI}}}$$
SUVBSADuBois	$$\frac{Radioactive concentration in tissue}{\frac{injected dose}{{\left(body weight\right)}^{0.425}*heigh{t}^{0.725}*0.007184}}$$
SUVBSAHaycock	$$\frac{Radioactive concentration in tissue}{\frac{injected dose}{{\left(body weight\right)}^{0.5378}*heigh{t}^{0.3964}*0.024265}}$$

*SUV* Standardized Uptake Value, *BW* Body Weight, *LBMJames* Lean Body Mass according to James, *LBMJanma* Lean Body Mass according to Janmahasatian, *BMI* Body Mass Index, *BSADuBois*, Body Surface Area according to Du Bois, *BMI* Body Mass Index, *BSAHaycock* Body Surface Area according to Haycock

### Statistical analysis

Descriptive statistics for categorical variables were presented as numbers with percentages. For continuous variables with normal distribution, means and standard deviations (SD) were provided, while medians and interquartile ranges (IQR) were used for non-normally distributed data. A two-sample t-test was performed to determine if there were differences in SUV measurements between male and female patients.

Scatterplots were created to visualise the relationship between body weight and mean SUV measurements for the different SUV formulations. A simple linear regression model was applied to these data, and the regression coefficients were used to assess the relative increase in SUV per kilogram, expressed as a/b from the equation:

1 $$\text{y}=\text{a x}+\text{b}$$

In this equation, a/b presents the relative SUV dependence on body weight, which can be readily compared between the different SUV formulations. The coefficient of determination (*r*^2^) was calculated to assess the degree of correlation between SUV measurements and body weight in the linear regression model.

Outliers were checked for potential data entry or measurement errors.

p-values less than 0.05 were considered significant. All analyses were performed using IBM SPSS Statistics version 29.0.0.0 and Python version 3.10.

### Ethical considerations

This study is retrospective and observational. The need for informed consent was waived by the institutional medical ethics committee. Data were pseudonymised and used solely for clinical research purposes.

## Results

### Patients

Between December 2018 and August 2022, 516 [^18^F]FDG PET/CT scans were acquired. Of those, a total of 461 scans were included in the study. Scans were excluded for the following reasons: multiple scans were taken on the same date from the same patient (n = 38), the presence of liver tumour or metastases (n = 14) and technical reasons (n = 3; n = 2 wrong dosage administrated and n = 1 unknown calibration time).

The study population consisted of 185 females (40%) and 276 males (60%). Median age at the time of the scan was 12 years, IQR 8 – 15.5 years. The median body height was 160 cm, IQR 132 – 172 cm, and the median body weight was 48.0 kg, IQR 27.7 – 60.8 kg (Table 2). Relations of age, body weight, height, LBM, and BSA are presented in supplemental Fig. 1.

**Table 2:** Patient characteristics

	All patients, *n* = 461
Age (years) median (IQR)	12 (8–15.5)
Height (cm) median (IQR)	160 (132 – 172)
Body weight (kg) median (IQR)	48 (27.7 – 60.8)
BMI (kg/m^2^) median (IQR)	18.3 (16.0 – 21.1)
LBMJames (kg) median (IQR)	39.6 (24.1 – 48.9)
LBMJanma (kg) median (IQR)	36.6 (23.6 – 47.4)
BSADuBois (m^2^) median (IQR)	1.48 (1.03 – 1.72)
BSAHaycock (m^2^) median (IQR)	1.46 (1.01 – 1.70)

*IQR* Interquartile range, *BMI* Body Mass Index, *LBMJames* Lean Body Mass according to James, *LBMJanma* Lean Body Mass according to Janmahasatian, *BSADuBois* Body Surface Area according to Du Bois, *BSAHaycock* Body Surface Area according to Haycock

Most patients were diagnosed with lymphoma (40%), followed by osteosarcoma (10%) and rhabdomyosarcoma (10%) (Table 3).

**Table 3:** Different tumour types

Tumour type	Number of patients (*n*)
Lymphoma	185
Osteosarcoma	48
Rhabdomyosarcoma	47
Ewing sarcoma	35
Non-rhabdomyosarcoma soft tissue sarcoma	26
Germ cell tumour	19
Leukaemia	15
Sarcoma (other)	10
Melanoma	8
Blastoma*	6
Benign tumor	5
Suspected malignancy, no diagnosis	26
Other**	29

*Tumour type ‘Blastoma’ contains pleuro pulmonary blastoma, sialoblastoma and lipoblastomatosis

** Tumour type ‘Other’ contains tumour types with a maximum of n = 2, found in the dataset. Tumour types include adenocarcinoma, lymphoepithelial carcinoma, adrenal cortical carcinoma, medullary carcinoma, renal cell carcinoma, squamous cell carcinoma, hematologic disease, sex cord stromal tumour, glioma, embryonal tumour, neuroblastoma, nephroblastoma and Langerhans cell histiocytosis

### SUV measurements

The median SUV measurements in the liver and blood pool, according to different SUV formulations, are presented in Tables 4 and 5. All SUV formulas show a significant difference between female and male with the exception of SUVLBMJames in both liver and blood pool, with p = 0.080 and p = 0.238, respectively, and of SUVBW in the liver, p = 0.057. SUVBW does show a significant difference between female and male in the blood pool, p = 0.046.

**Table 4:** SUV in liver, according to different formulations in all patients, for female patients, and for male patients

Liver SUV	All patients, *n* = 461	Female, *n* = 185	Male, *n* = 276	*p*-value
SUVBW median (IQR)	1.48 (1.17 – 1.77)	1.54 (1.17 – 1.84)	1.44 (1.17 – 1.72)	0.057
SUVLBMJames median (IQR)	1.22 (1.01 – 1.40)	1.20 (0.96 – 1.37)	1.24 (1.04 – 1.45)	0.080
SUVLBMJanma median (IQR)	1.16 (0.97 – 1.35)	1.07 (0.84 – 1.22)	1.25 (1.06 – 1.43)	** < 0.001**
SUVBSADuBois median (IQR)	0.046 (0.042 – 0.051)	0.048 (0.042 – 0.053)	0.045 (0.041 – 0.050)	** < 0.001**
SUVBSAHaycock median (IQR)	0.046 (0.041 – 0.050)	0.048 (0.042 – 0.053)	0.045 (0.040 – 0.049)	** < 0.001**

*SUV* Standardized Uptake Value, *IQR* Interquartile range, *BW* Body Weight, *LBMJames* Lean Body Mass according to James, *LBMJanma* Lean Body Mass according to Janmahasatian, *BSADuBois* Body Surface Area according to Du Bois, *BSAHaycock* Body Surface Area according to Haycock. *p* < 0.05 was considered significant

**Table 5:** SUV in blood pool, according to different formulations in all patients, for female patients, and for male patients

Blood pool SUV	All patients, *n* = 461	Female, *n* = 185	Male, *n* = 276	*p*-value
SUVBW median (IQR)	1.13 (0.88 – 1.37)	1.19 (0.90 – 1.42)	1.10 (0.87 – 1.33)	**0.046**
SUVLBMJames median (IQR)	0.95 (0.76 – 1.10)	0.94 (0.74 – 1.08)	0.95 (0.77 – 1.13)	0.238
SUVLBMJanma median (IQR)	0.90 (0.74 – 1.07)	0.83 (0.65 – 0.95)	0.96 (0.78 – 1.13)	** < 0.001**
SUVBSADuBois median (IQR)	0.036 (0.031 – 0.0.40)	0.038 (0.033 – 0.042)	0.035 (0.030 – 0.040)	** < 0.001**
SUVBSAHaycock median (IQR)	0.035 (0.031 – 0.040)	0.038 (0.032 – 0.042)	0.034 (0.030 – 0.039)	** < 0.001**

*SUV* Standardized Uptake Value, *IQR* Interquartile range, *BW* Body Weight, *LBMJames* Lean Body Mass according to James, *LBMJanma* Lean Body Mass according to Janmahasatian, *BSADuBois* Body Surface Area according to Du Bois, *BSAHaycock* Body Surface Area according to Haycock. *p* < 0.05 was considered significant

### Correlation with body weight

**Fig. 1:**
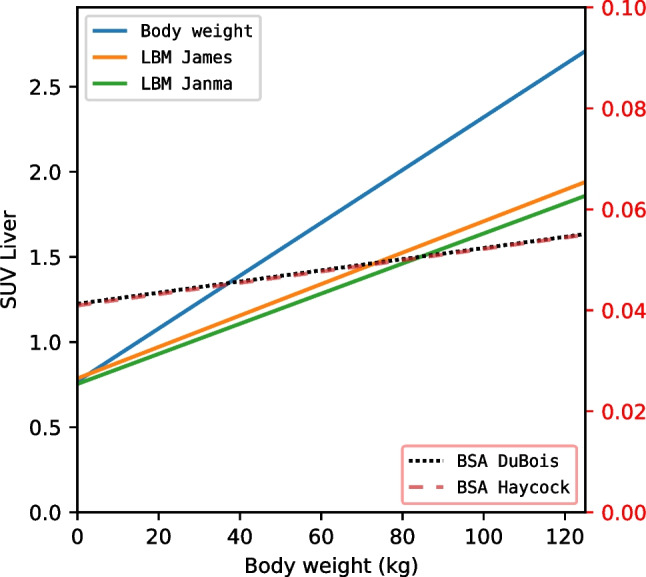
Regression lines of SUV measured in the liver corrected for body weight, corrected for LBM according to James, corrected for LBM according to Janma, corrected for BSA according to DuBois, and corrected for BSA according to Haycock. Left y-axis shows SUV for body weight, LBM according to James, and LBM according to Janma. Right y-axis shows SUV for BSA according to DuBois, and BSA according to Haycock. LBM James, Lean Body Mass according to James; LBM Janma, Lean Body Mass according to Janmahasatian; BSA DuBois, Body Surface Area according to Du Bois; BSA Haycock, Body Surface Area according to Haycock

**Fig. 2:**
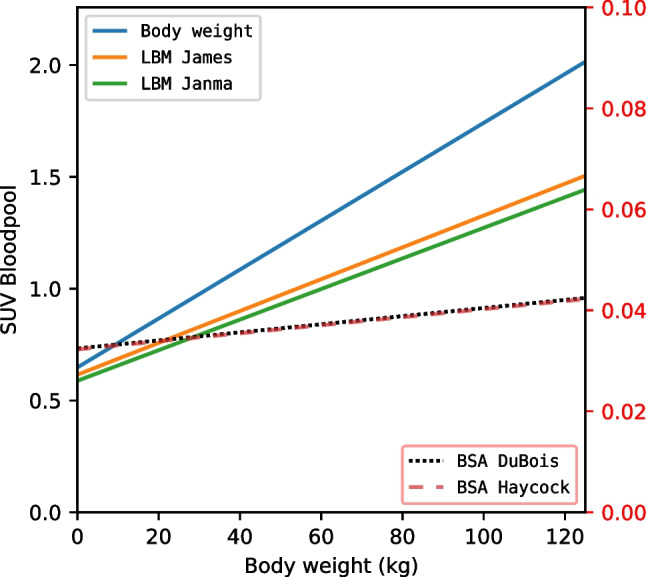
Regression lines of SUV measured in the blood pool corrected for body weight, LBM according to James, LBM according to Janma, BSA according to DuBois, and BSA according to Haycock. Left y-axis shows SUV for body weight, LBM according to James, and LBM according to Janma. Right y-axis shows SUV for BSA according to DuBois, and BSA according to Haycock. LBM James, Lean Body Mass according to James; LBM Janma, Lean Body Mass according to Janmahasatian; BSA DuBois, Body Surface Area according to Du Bois; BSA Haycock, Body Surface Area according to Haycock

As expected, based on the SUV formulation, all showed positive correlations with body weight. Figures 1 and 2, Tables 6 and 7, and supplemental Fig. 2 and 3, show that SUVBW has the highest correlation with body weight with *r*^2^ = 0.65 and *r*^2^ = 0.50, measured in liver and blood pool respectively. SUVBSADuBois and SUVBSAHaycock have the weakest correlations which is the similar for both formulations, *r*^2^ = 0.09 for the liver and *r*^2^ = 0.06 for the blood pool. SUVLBMJames has a correlation *r*^2^ = 0.51 and *r*^2^ = 0.44, measured the liver and blood pool respectively, and SUVLBMJanma has a correlation *r*^2^ = 0.47 and *r*^2^ = 0.42, measured in the liver and blood pool respectively.

**Table 6:** Relation of SUV and body weight measured in the liver, presented as linear regression coefficients, the relative SUV dependence on body weight (a/b), and the coefficient of determination (*r*^2^)

Liver SUV		All patients, *n* = 461	Female, *n* = 185	Male, *n* = 276
SUVBW	Linear regression coefficients	y = 0.77 + 15.5E-3x	y = 0.77 + 17.1E-3x	y = 0.75 + 14.9E-3x
	a/b	0.0260	0.0260	0.0133
	*r*^2^	0.65	0.65	0.68
SUVLBMJames	Linear regression coefficients	y = 0.79 + 9.23E-3x	y = 0.79 + 8.97E-3x	y = 0.79 + 9.33E-3x
	a/b	0.0117	0.0114	0.0118
	*r*^2^	0.51	0.44	0.54
SUVLBMJanma	Linear regression coefficients	y = 0.75 + 8.84E-3x	y = 0.83 + 8.73E-3x	y = 0.68 + 8.34E-3x
	a/b	0.0118	0.0105	0.0123
	*r*^2^	0.47	0.48	0.50
SUVBSADuBois	Linear regression coefficients	y = 0.04 + 1.11E-4x	y = 0.04 + 1.18E-4x	y = 0.04 + 1.14E-4x
	a/b	0.0028	0.0030	0.0029
	*r*^2^	0.09	0.08	0.12
SUVBSAHaycock	Linear regression coefficients	y = 0.04 + 1.12E-4x	y = 0.04 + 1.20E-4x	y = 0.04 + 1.17E-4x
	a/b	0.0028	0.0030	0.0043
	*r*^2^	0.09	0.08	0.12

*SUV* Standardized Uptake Value, *BW* Body Weight, *LBMJames* Lean Body Mass according to James, *LBMJanma* Lean Body Mass according to Janmahasatian, *BSADuBois* Body Surface Area according to Du Bois, *BSAHaycock* Body Surface Area according to Haycock

**Table 7:** Relation of SUV and body weight measured in the blood pool, presented as linear regression coefficients, the relative SUV dependence on body weight (a/b), and the coefficient of determination (*r*^2^)

Blood pool SUV		All patients, *n* = 461	Female, *n* = 185	Male, *n* = 276
SUVBW	Linear regression coefficients	y = 0.65 + 10.9E-3x	y = 0.75 + 10.0E-3x	y = 0.57 + 11.7E-3x
	a/b	0.0153	0.0133	0.0175
	*r*^2^	0.50	0.36	0.64
SUVLBMJames	Linear regression coefficients	y = 0.62 + 7.12E-3x	y = 0.64 + 6.53E-3x	y = 0.60 + 7.44E-3x
	a/b	0.0115	0.0102	0.0124
	*r*^2^	0.44	0.35	0.49
SUVLBMJanma	Linear regression coefficients	y = 0.59 + 6.84E-3x	y = 0.55 + 6.09E-3x	y = 0.63 + 6.97E-3x
	a/b	0.0116	0.0111	0.0111
	*r*^2^	0.42	0.39	0.46
SUVBSADuBois	Linear regression coefficients	y = 0.03 + 8.01E-5x	y = 0.03 + 6.73E-5x	y = 0.03 + 9.49E-5x
	a/b	0.0027	0.0022	0.0032
	*r*^2^	0.06	0.03	0.11
SUVBSAHaycock	Linear regression coefficients	y = 0.03 + 7.97E-5x	y = 0.03 + 6.67E-5x	y = 0.03 + 9.52E-5x
	a/b	0.0027	0.0022	0.0032
	*r*^2^	0.06	0.03	0.11

*SUV* Standardized Uptake Value, *BW* Body Weight, *LBMJames* Lean Body Mass according to James, *LBMJanma* Lean Body Mass according to Janmahasatian, *BSADuBois* Body Surface Area according to Du Bois, *BSAHaycock* Body Surface Area according to Haycock

The relative SUV dependence on body weight, a/b in Eq. 1, is lowest in both SUVBSADuBois and SUVBSAHaycock, with a/b = 0.003, which is the same in liver and blood pool. SUVLBMJames has dependence of a/b = 0.012, similar in both liver and blood pool, which is the same for SUVLBMJanma. SUVBW has the highest dependence on body weight with a/b = 0.026 and a/b = 0.015, in liver and blood pool respectively.

## Discussion

SUV measurements are not yet a standard part of routine [^18^F]FDG PET/CT interpretation in paediatric oncology, primarily being employed in research settings. However, metrics such as metabolic tumour volume (MTV) and total lesion glycolysis (TLG), along with SUV measurements, are increasingly utilized in research protocols to evaluate tumour burden and monitor treatment response. In paediatric oncology, understanding weight dependency is particularly relevant due to the significant variability in body composition among children of different ages and developmental stages.

This study aimed to compare different SUV formulations in paediatric oncology patients and assess their dependency on body weight. In a large dataset of 461 [^18^F]FDG PET/CT scans, our findings confirm that SUVBW exhibits the highest dependency on body weight compared to the SUVLBM formulations (SUVLBMJames and SUVLBMJanma) and the SUVBSA formulations (SUVBSADuBois and SUVBSAHaycock). These results are consistent with previous studies involving adults and a small paediatric population, which have demonstrated that SUVLBM and SUVBSA adjustments reduce weight dependency in SUV measurements [1, 2, 7, 17].

Use of different SUV metrics has been extensively validated against full kinetic analysis in adult populations, such as the studies by Hoekstra et al. and Freedman et al. [18, 19]. These studies highlighted the importance of aligning SUV calculations with physiological considerations to improve accuracy and reduce variability. Although these validations were performed in adults rather than children, they provide important context for the current findings, as we observe similar patterns of reduced weight dependency with SUVLBM and SUVBSA formulations. Our findings align with these earlier observations, further supporting the broader utility of SUVLBM as a robust metric in [^18^F]FDG PET imaging.

Among the different SUV formulations studied, SUVBSADuBois and SUVBSAHaycock showed the lowest dependency on body weight. For example, based on our dataset, the SUVBW in the liver varied significantly between a child weighing 10 kg and one weighing 40 kg, with SUVs of 0.97 and 1.57, respectively, a difference by a factor of 1.62. In contrast, SUVBSAHaycock varied only slightly, by a factor of 1.08, between the same two children, with SUVs of 0.04112 and 0.04448, respectively. These findings suggest that SUVBSA formulations may provide more reliable measurements in paediatric patients, particularly when body weight varies considerably.

A major strength of this study is the inclusion of a large cohort of 461 paediatric patients, all of whom were scanned on the same [^18^F]FDG PET/CT system. This consistency, combined with the wide range of patient ages, enhances the generalisability of our findings to the broader paediatric population.

However, several limitations should be acknowledged. First, the restriction to the first [^18^F]FDG PET/CT scan during the study period precluded an analysis of how SUV measurements change over time in individual patients. This could be an important consideration in assessing treatment response. Second, since all SUV formulations incorporate body weight to some degree, the correlation with body weight was never fully eliminated, limiting the independence of these correlations. Third, we did not account for the potential influence of factors like glucose and tumour load. It is known that these factors can influence SUV as they influence the distribution of [^18^F]FDG throughout the body. Last, we did not account for the potential influence of chemotherapy administered to an unknown number of patients before the [^18^F]FDG PET/CT scan, which may have impacted SUV measurements, particularly in the liver. However, we mitigated this limitation by also measuring SUV in the blood pool, where chemotherapy has minimal influence, to serve as a control and ensure robustness of our findings.

Our findings have important clinical implications for paediatric [^18^F]FDG PET/CT imaging. Given the strong dependency of SUVBW on body weight and its tendency to inflate values in heavier patients, this formulation should be avoided in routine clinical practice. Both SUVLBM and SUVBSA formulations offer lower dependency on body weight and thus provide more consistent assessments across different patient groups. While the equations for LBM and BSA are empirically derived and widely validated for adults, their applicability in paediatric patients should be carefully considered. The BSAHaycock formulation, specifically developed and validated for children, is particularly suited for paediatric applications and has also been widely adopted in pharmacokinetics due to its geometric basis and broad applicability across ages [6]. Nonetheless, these equations may not accurately represent body composition in extreme cases, such as top athletes or patients with atypical body proportions, which could limit their generalizability in certain context. Interestingly, BSA appears to perform better than LBM as a normalisation factor in this paediatric population, likely due to differences in [^18^F]FDG clearance mechanisms between children and adults. In adults, only up to 10% of [^18^F]FDG is excreted via urine within 60 min post-injection, whereas in children, kidney clearance and the correlation of BSA with cardiac output and blood volume may play a larger role in [^18^F]FDG distribution. Additionally, the percentage of body fat in children fluctuates considerably, reaching up to 25% during the first year of life, which may limit the accuracy of LBM as a surrogate. While LBM is a good indicator of liver metabolism, its practical applicability appears less robust in children compared to BSA.

Normalisation metrics like body weight, LBM, and BSA aim to approximate the bioavailability of [^18^F]FDG from blood to tissues by accounting for factors such as blood volume and clearance mechanisms. Body weight normalisation is simple but fails to consider body composition variability. LBM improves upon this by excluding adipose tissue, but fluctuating fat percentages in children limit its reliability. BSA, with its strong correlation to cardiac output and blood volume, provides a better surrogate for [^18^F]FDG bioavailability in paediatric patients [20, 21].

It is also worth noting that SUVBSA is not unitless, as BSA is expressed in m^2^. This contrasts with the SUVLBM and SUVBW formulations, which can be converted to a volumetric unit by dividing by tissue density. Consequently, SUVBSA values are generally lower, complicating direct comparisons with SUVLBM and SUVBW.

Furthermore, from a practical standpoint, it would be prudent to adhere to the PERCIST (PET Response Criteria in Solid Tumours) guidelines, which recommend the utilisation of SUVLBM for the assessment of both adult and paediatric patients [22], and based on our findings, SUVLBMJames may be a particularly favourable formulation. It’s worth noting that both SUVLBMJames and SUVLBMJanma have gender specific formulations (Table 1). Unlike other formulations, SUVLBMJames did not exhibit significant differences between male and female patients, suggesting it to be a more gender-neutral option for clinical use. This is an important consideration for ensuring consistent and unbiased SUV measurements in paediatric populations.

Looking forward, advancements in imaging technology could further improve the accuracy of SUV measurements in paediatric patients. Dual-energy CT or machine learning-based body composition analysis offers the potential to provide more accurate estimates of lean body mass and body composition [23, 24]. These technologies could support better quantification of [^18^F]FDG uptake by providing more individualised adjustments to SUV calculations, minimising the dependency on crude body weight measures and potentially leading to even more accurate and reliable assessments of metabolic activity.

## Conclusion

In paediatric patients undergoing [^18^F]FDG PET/CT scans, SUVBW should be avoided due to its dependency on body weight and the elevated values it produces in heavier patients. SUVBSA presents the least dependency on body weight and provides the most consistent assessments of metabolic activity.

## Supplementary Information

Below is the link to the electronic supplementary material.

Supplementary file1 (DOCX 1070 KB)

## Data Availability

The datasets generated and analyzed during the current study are available from the corresponding author upon reasonable request.
