# Supplementary material for: Improving accuracy of SUV estimates in paediatric oncology: Recommending against the use of body weight corrected SUV in [18F]FDG PET
Source: Eur J Nucl Med Mol Imaging. 2025 Feb 8;52(7):2444–51. doi: 10.1007/s00259-025-07104-6 (PMC12119731; doi:10.1007/s00259-025-07104-6)
Supplement: Supplementary file 1 — Supplementary file1 (DOCX 1070 KB) [file 259_2025_7104_MOESM1_ESM.docx]

**Supplemental data**

**Table 1.** Patients characteristics in all patients, for female patients, and for male patients.

|  | **All patients, n = 461** | **Female, n = 185** | **Male, n = 276** | **p-value** |
| --- | --- | --- | --- | --- |
| **Age (years)** median (IQR) | 12.0 (8.0 – 15.5) | 13.0 (8.0 – 16.0) | 12.0 (8.0 – 15.0) | 0.627 |
| **Height (cm)** median (IQR) | 160 (132 – 172) | 159 (131 – 168) | 162 (133 – 176) | **0.009** |
| **Body weight (kg)** median (IQR) | 48.0 (27.7 – 60.8) | 48.0 (28.0 – 58.4) | 48.0 (27.4 – 63.5) | 0.136 |
| **BMI (kg/m^2^)** median (IQR) | 18.3 (16.0 – 21.1) | 18.3 (16.0 – 21.2) | 18.2 (16.0 – 20.7) | 0.581 |
| **LBMJames (kg)** median (IQR) | 39.6 (24.1 – 48.9) | 38.2 (23.1 – 43.8) | 41.5 (25.0 – 52.5) | **<0.001** |
| **LBMJanma (kg)** median (IQR) | 36.6 (23.6 – 47.4) | 33.5 (20.4 – 38.5) | 41.7 (25.5 – 52.2) | **<0.001** |
| **BSADuBois (m^2^)** median (IQR) | 1.48 (1.03 – 1.72) | 1.48 (1.03 – 1.65) | 1.48 (1.04 – 1.77) | 0.057 |
| **BSAHaycock (m^2^)** median (IQR) | 1.46 (1.01 – 1.70) | 1.46 (1.01 – 1.63) | 1.47 (1.01 – 1.74) | 0.086 |

IQR, Interquartile range; BMI, Body Mass Index; LBMJames, Lean Body Mass according to James; LBMJanma, Lean Body Mass according to Janmahasatian; BSADuBois, Body Surface Area according to Du Bois; BSAHaycock, Body Surface Area according to Haycock. p-values < 0.05 were considered significant.

**Figure 1.** Relations of age, height, body mass index, body weight, lean body mass according to James, lean body mass according to Janmahasatian, body surface area according to Du Bois, and body surface area according to Haycock.


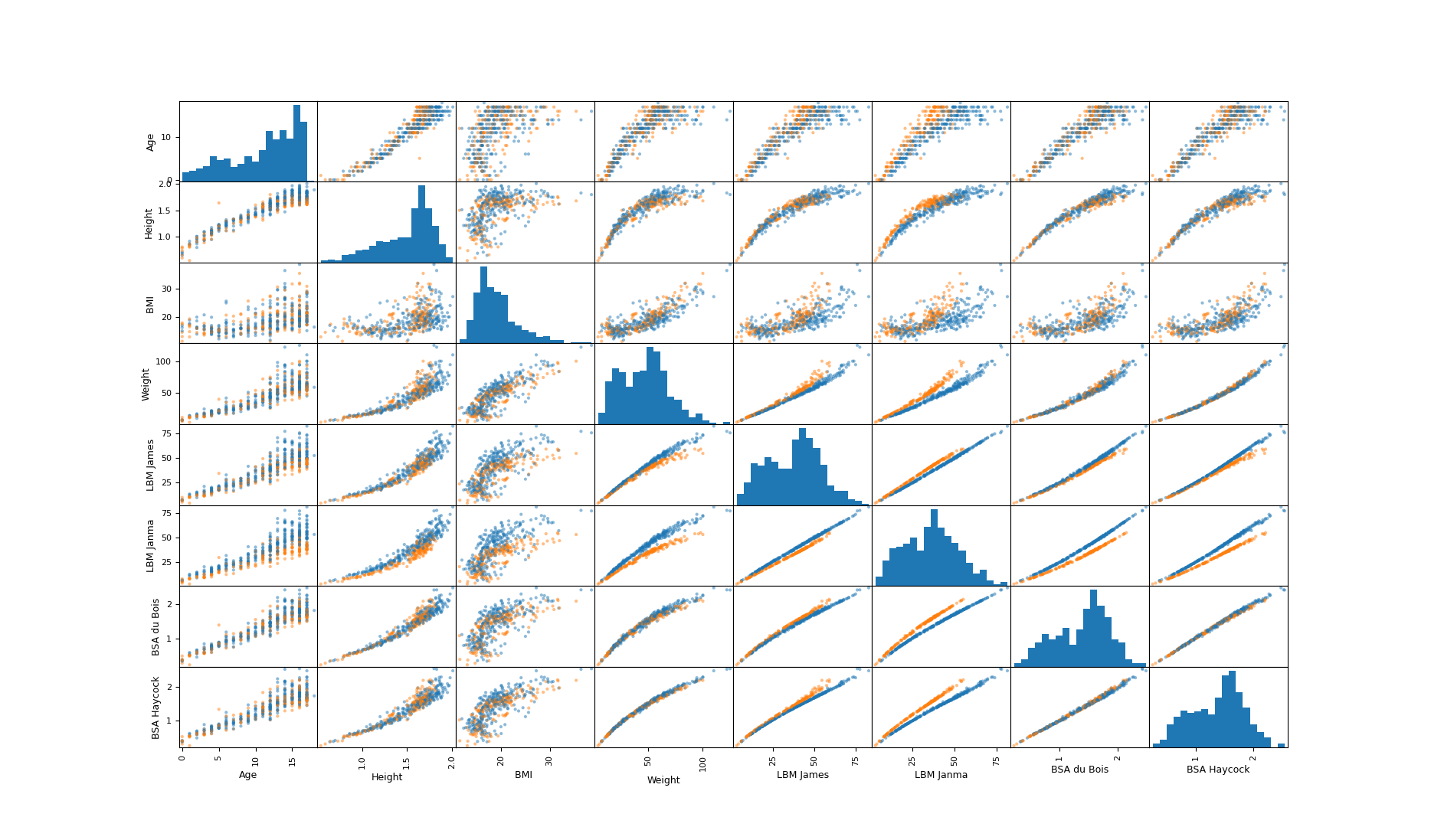


BMI, Body Mass Index; LBM James, Lean Body Mass according to James; LBM Janma, Lean Body Mass according to Janmahasatian; BSA DuBois, Body Surface Area according to Du Bois; BSA Haycock, Body Surface Area according to Haycock


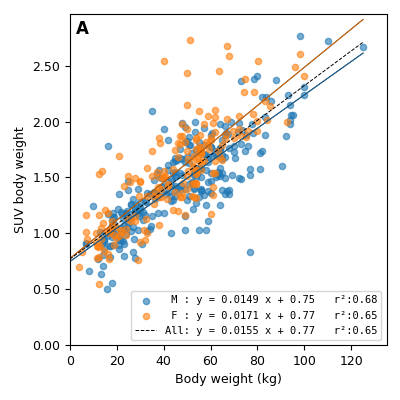


**Figure 2A-E** SUV measured in liver and body weight with Eq. 1 and *r*^2^ for male, female, and all patients. **A** SUV corrected for body weight. **B** SUV corrected for LBM according to James. **C** SUV corrected for LBM according to Janma. **D** SUV corrected for BSA according to DuBois. **E** SUV corrected for BSA according to Haycock.


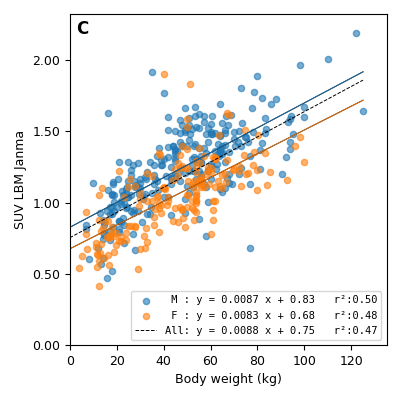

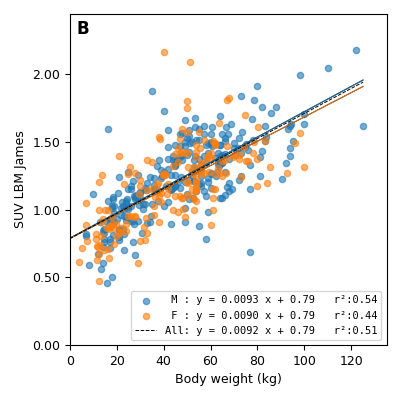


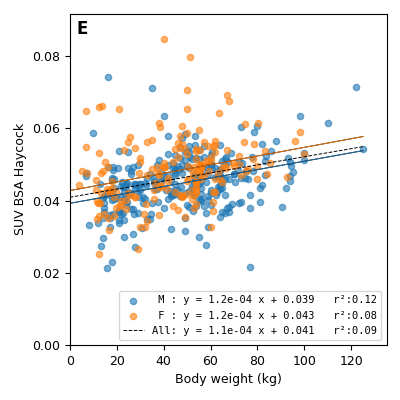

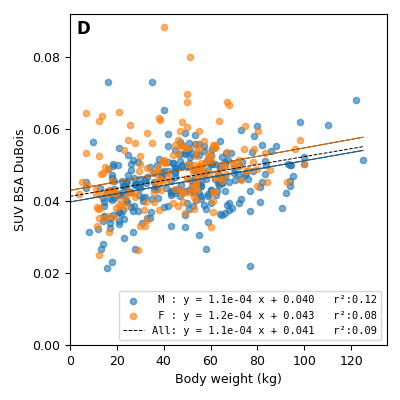


SUV, Standardized Uptake Value; LBM James, Lean Body Mass according to James; LBM Janma, Lean Body Mass according to Janmahasatian; BSA DuBois, Body Surface Area according to Du Bois; BSA Haycock, Body Surface Area according to Haycock; M, male; F, female.


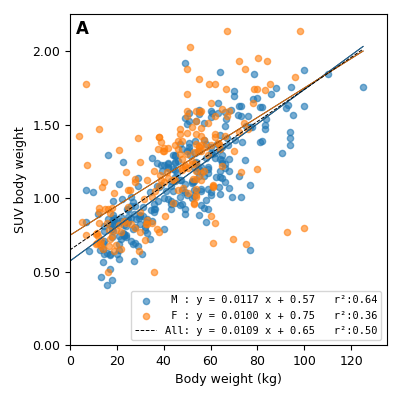


**Figure 3A-E** SUV measured in blood pool and body weight with Eq. 1 and *r*^2^ for male, female, and all patients. **A** SUV corrected for body weight. **B** SUV corrected for LBM according to James. **C** SUV corrected for LBM according to Janma. **D** SUV corrected for BSA according to DuBois. **E** SUV corrected for BSA according to Haycock.


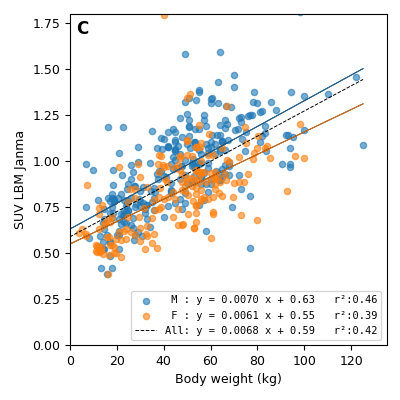

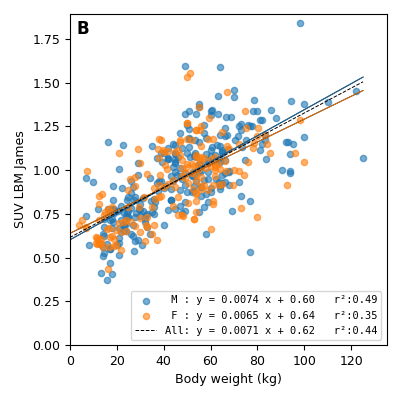


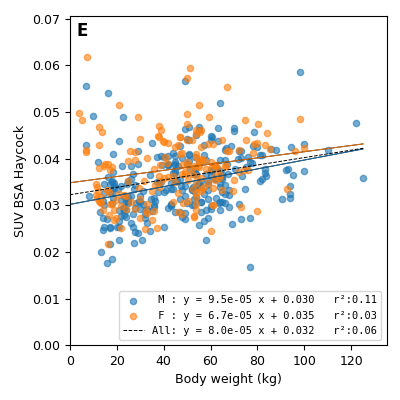

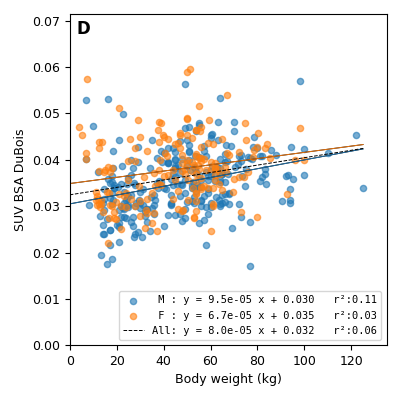


SUV, Standardized Uptake Value; LBM James, Lean Body Mass according to James; LBM Janma, Lean Body Mass according to Janmahasatian; BSA DuBois, Body Surface Area according to Du Bois; BSA Haycock, Body Surface Area according to Haycock; M, male; F, female.
